# A haven of green space: learning from a pilot pre-post evaluation of a school-based social and therapeutic horticulture intervention with children

**DOI:** 10.1186/s12889-018-5661-9

**Published:** 2018-07-05

**Authors:** Anna Chiumento, Ipshita Mukherjee, Jaya Chandna, Carl Dutton, Atif Rahman, Katie Bristow

**Affiliations:** 1 Institute of Psychology, Health and Society, University of Liverpool, Waterhouse Building Block B Floor 2, Liverpool, L69 3GL UK; 2 Division of Psychology and Mental Health, University of Manchester, Oxford Rd, Manchester, M13 9PL UK; 3 Institute of Transitional Medicine, University of Liverpool, Alder Hey Children’s Hospital, Eaton Rd, Liverpool, L14 5AB UK; 4 Child and Adolescent Mental Health, Alder Hey Children’s Hospital, Eaton Rd, Liverpool, L14 5AB UK; 5 Institute of Psychology, Health and Society, University of Liverpool, Waterhouse Building Block B Floor 1, Liverpool, L69 3GL UK

**Keywords:** Children and young people, Greenspace, Behavioural, emotional and social problems, Mental health and psychosocial wellbeing, Schools, Community-based, Therapeutic horticulture, Mixed-methods, Pilot intervention

## Abstract

**Background:**

Research suggests outdoor activity in green spaces is important for children’s mental, emotional and social wellbeing. A recognised green space intervention is “Social and Therapeutic Horticulture” (STH). We discuss findings from a pilot STH intervention, “A Haven of Green Space” conducted in North West England. The target group were school children aged 9–15 years experiencing behavioural, emotional and social difficulties. This exploratory study aims to assess the mental wellbeing of the children pre- and post-intervention, and assess the value of the evaluation methods and “Five Ways to Wellbeing” evaluation framework.

**Methods:**

The intervention involved 6 monthly sessions with two horticulturists and a psychotherapist. Sessions were participatory with the development of selected greenspaces at each school directed by the children. Evaluation was situated in the “Five Ways to Wellbeing” framework, using a mixed-methods pre- post-evaluation design. Existing public mental health evaluation methodologies were adapted for use with school children: Mental Well Being Impact Assessment (MWIA) and Wellbeing Check Cards. The MWIA was analysed qualitatively identifying over-arching themes. The quantitative wellbeing check cards were analysed by mean score comparison.

**Results:**

Results were collected from 36 children across the three participating schools, and suggest that the Haven Green Space intervention was associated with improved mental wellbeing. MWIA factors relating to mental wellbeing (“emotional wellbeing” and “self-help”) were positively impacted in all three schools. However, findings from the wellbeing check cards challenge this, with worsening scores across many domains.

**Conclusions:**

A key study limitation is the pilot nature of the intervention and challenges in adapting evaluation methods to context and age-range. However, results indicate that group based socially interactive horticulture activities facilitated by trained therapists are associated with positive impacts upon the mental and emotional wellbeing of children experiencing behavioural, emotional and social difficulties. Further research is needed to verify this, and to support using the “Five Ways” in intervention development and evaluation. Finally, we recommend continued efforts to develop age-appropriate evaluation methods.

## Background

Access to green space and nature is recognised to improve the mental wellbeing of children [1, 2]. Green spaces are considered to have restorative and relaxing properties enabling an ‘escape’ from urban living, in addition to providing spaces for exercise [3–7]. Research demonstrates that the quality of the natural environment positively affects the personal development of children and young people [hereafter referred to collectively as “children”] [8–10]. However, there are concerns about children becoming increasingly distanced from the natural environment [2, 11, 12], with reasons for this suggested to include new technologies, safety concerns, and the reduction in quality urban environments [2, 13–15].

Educational and ecological research suggest that outdoor nature play is important for children’s mental, emotional and social wellbeing by developing connections to social and physical environments whilst stimulating imagination and creativity [10, 16]. Three theories seek to explain the impact of connections to nature upon mental health and wellbeing: Biophilia [17], Stress Reduction Theory [18] and Attention Restoration Theory [19]. The latter two are of therapeutic interest. Stress Reduction Theory [18] suggests that particular environments produce certain effects, with perceived “safe” environments triggering positive emotional responses. Attention Restoration Theory [19] proposes that nature assists with recovery from attention fatigue, allowing distance from routine activities and thoughts to engage without conscious effort.

The Children’s Environment and Health Action Plan for Europe includes a commitment to improving child wellbeing through promotion of physical activity and access to green spaces [9], acknowledging the therapeutic benefit of physical activity in green spaces. One form of green space activity recognised for its social and therapeutic benefit across different population groups is horticulture [20, 21]. Sempik uses the term “Social and Therapeutic Horticulture” (STH) to describe greenspace-based interventions with vulnerable groups such as those with mental health needs [20–24]. A core feature of Sempik and colleagues is a focus on improving the wellbeing of participants engaged in STH, rather than productive gardening [20, 22, 25, 26], in line with Stress Reduction and Attention Restoration theories. Others [27–31], also identify the positive impacts of greenspace and horticulture-based interventions on the mental wellbeing of children.^1^

This paper discusses findings of a pilot STH intervention, “A Haven of Green Space” (hereafter “Haven Green space”), alongside methodological reflections. Recognising that the terminology surrounding space and place is contested and varies by discipline (for a comprehensive review, see [33]), for the purpose of this paper we follow Cresswell [34] in adopting a broad definition whereby the “Haven of Green Space” represents place as “a meaningful location” ([34] p12). This definition of place entails three elements of [1] a location, [2] a locale or material setting for interactions, and [3] evoking subjective and emotional attachments [35]. Therefore, the Haven of Green Space project draws upon both the physical and social connotations of the term “place” [33] as engagement with school green spaces involved moving from green space as a blank canvas, to the creation of place through group psychotherapeutic activities such as the everyday practice of caring for nature, the production of art or symbols, and the creation of names, local ceremonies, and myths attached to specific places – all recognised as place-making activities [34].

This project was funded by a Primary Care Trust (PCT, since 2012 a Clinical Commissioning Group) and a City Council in the North West of England as part of a large-scale health and wellbeing grants programme. An important funding requirement was to situate the intervention evaluation within the “Five Ways to Wellbeing” framework (hereafter “Five Ways”) whose central tenant is to tackle health inequalities through promoting physical and mental health [36]. The Five Ways actions seek to influence mental wellbeing by playing an essential role in satisfying needs for positive relationships, personal autonomy, competency and security [36]. As an action oriented approach, the Five Ways aim to promote behaviour change by creating feedback loops that encourage people to reflect upon and adopt behaviours that promote wellbeing [36]. There is an expanding literature on intervention studies which contribute to the evidence-base for the Five Ways [36]. Although developed for adults, the Five Ways has been validated with children, with slight modifications of language to ensure age-appropriateness [37].

### Haven green space pilot intervention

The Haven Green Space intervention involved monthly sessions over 6 months in which the participating children designed a green space facilitated by two horticulturists and a Child and Adolescent Mental Health Service (CAMHS) psychotherapist. Each session was two hours long and employed STH and psychotherapeutic techniques to facilitate exploration of environmental and wellbeing themes in line with the five ways. Table 1 illustrates how the five ways are applied within the Haven Green Space intervention.

**Table 1:** Application of the Five Ways in Haven Green space intervention

Five Ways Action	Application in Haven Green Space
Connect: to those around you and to the natural environment	• Children engaging in shared activities in pairs and full groups. • Taking care of and connecting to the school’s green space environment, including recognising areas that lacked greenery and working to improve this. • Connecting with others outside of the Haven Green Space group, for example engaging in activities such as planting or socialising in green spaces such as parks.
Be active: engage in enjoyable physical activity	• Physical activity linked to horticulture e.g. digging, planting, watering etc. • Painting and decorating the green space.
Take Notice: of the world around you and of your feelings	• Being outside facilitates noticing changing seasons and growth of plants / development of the green space. • Working with others in a shared green space necessitates team work and negotiation and is an interaction that encouraged awareness of one’s own and others feelings. • Reporting positive and negative interactions with green spaces outside of the group, i.e. planting with family members or friends, or being unable to access green space due to their use by older children perceived as bullies.
Keep Learning: to build confidence and have fun	• Opportunities for learning horticultural skills such as planting and nurturing plants. • Learning about how to manage both success and failure when growing plants. • Engaging in spontaneous play within the green space.
Give: do something nice for a friend or stranger, linking with the wider community	• Developing a green space for others to enjoy. • Planning for the future of the green space as a legacy for the school. • Sharing what they have been growing in Haven Green Space group with teachers and fellow pupils including taking into class plants that had been grown or items discovered in the green space such as stones or broken pottery. • Applying skills learnt in the green space to other opportunities for engagement with nature e.g. growing plants at home.

Haven Green space was supported and steered by an advisory group comprised of academic researchers and experts from the fields of public health, child and adolescent primary mental health care, and education. Advisory group meetings were attended by the research and intervention teams every six weeks to review progress and critically reflect on emerging findings and challenges.

## Methods

### Pilot evaluation setting

The Haven Green space intervention targeted school children experiencing behavioural, emotional and social difficulties (BESD) in three schools in a city in the North West England. Estimates for BESD vary considerably, not least because what constitutes BESD is disputed, making the calculation of prevalence difficult. Visser [38] estimates that between 10 and 20% of 4–16 year olds in England experience some degree of BESD that interrupts their social and emotional development. These can lead to psychiatric disorders in later life [39, 40]. Haven Green Space aimed to promote positive mental, emotional and physical wellbeing of the children taking part. The intervention evolved out of a similar horticulture project at a secondary school that proved popular and appeared to deliver positive benefits. For Haven Green Space it was decided to pilot the approach in primary as well as secondary schools to reach a wider age range of children.

At the time of the study the ward in which the schools are located suffered high levels of deprivation, as measured by the Indices of Multiple Deprivation compiled by the Government [41] . The ward frequently fell within the 5% most deprived areas of the county, including child poverty rates of over 45% - more than double the national average [41]. The ward has been identified as an area in need of green infrastructure expansion and early intervention educational programmes to encourage healthy behaviours [42]. Across the schools at the time of intervention delivery there was an average of 16% of pupils with a Special Educational Needs (SEN) statement, and around 26% of children for whom English was not their first language [43–45]. The secondary school suffered high rates of absenteeism, almost double the average for English state funded secondary schools [46].

### Participants

The intervention took place in two primary schools (Schools A and B) and one secondary school (School C) between February and July 2012. These schools were selected as pupils from the primary schools automatically have a place at the secondary school, and existing relationships between the schools and the CAMH therapist involved in intervention delivery was felt to increase opportunities for successful intervention implementation and evaluation.

Haven Green space targeted children in years five and six in the primary schools (aged 9–12), and years seven to nine in the secondary School (aged 12–15). The decision to work with children of a similar age was to ensure similar levels of maturity and educational understanding of the environment and wellbeing. The sampling approach was purposive, with teachers from each school inviting children who had been identified as experiencing BESD through the schools’ Pastoral Care Programme to participate in the intervention. This approach sought to remain as close as possible to the anticipated referral process in the natural school setting should the service be adopted into mainstream support services. Therefore, identification and referral was dependent upon the Pastoral Care Programme’s knowledge of and engagement with children experiencing difficulties. This approach entailed a conscious decision not to impose identification of children based upon mental health or emotional wellbeing screening tools which would be unlikely to form part of routine practice. Children known to be undergoing treatment with CAMHS were excluded.

### Evaluation process

#### Evaluation aims

The primary aim of the evaluation was to assess whether there were any trends in the mental health and wellbeing of the participating children. In addition, given the central role of the Five Ways, we wanted to explore how existing evaluation and outcome measures might assess the Five Ways in the context of children’s wellbeing. To enable this two existing public mental health evaluation methodologies were adapted for use with school children: the Mental Well Being Impact Assessment [47] and Wellbeing Check Cards [48]. As such, the purpose of this paper is to not only to discuss the evaluation findings, but also to critically reflect on the evaluation approach.

##### Evaluation design

This exploratory evaluation followed recommendations to conduct small-scale pilot studies [49]. As such, it was not powered to assess intervention effectiveness but verify the feasibility of implementing novel interventions, and assess the suitability of research tools and procedures [49]. To achieve this a mixed-methods triangulation evaluation design was employed ([50] p62) involving the use of qualitative and quantitative methods used concurrently to gather complementary exploratory data relating to the impact of Haven Green Space upon the mental wellbeing of participating children. In order to assess the primary outcome of children’s wellbeing the Mental Wellbeing Impact Assessment (MWIA) [47] was administered pre- and post-intervention, and the Wellbeing Check Cards [48] - based upon the 7-item Warwick-Edinburgh Mental Well-being Scale (WEMWBS) [51, 52] - were administered at the first and last intervention session. Other methods used include interviewing teachers in each school at the end of the intervention, and asking teachers to complete pre- and post-Strengths and Difficulties Questionnaire for participating pupils [53, 54]; a child self-reporting questionnaire hosted by Puzzled Out^2^ [55]; and Draw and Write journals [56]. This paper does not discuss the teacher SDQs or qualitative interviews, or the latter two child focussed methods.

### Adaptation and application of evaluation tools for data collection

#### Qualitative methods

##### Mental wellbeing impact assessment

**Fig. 1:**
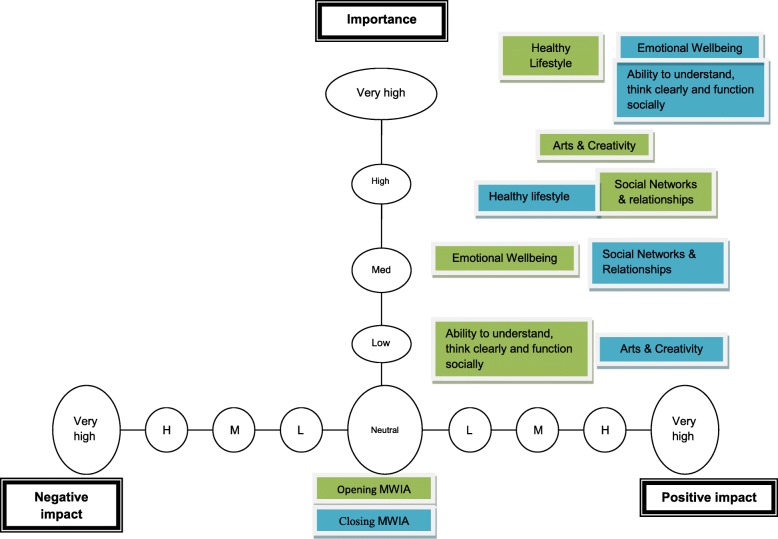
Example grid for domain increasing resilience and community assets from School C

The Mental Well-being Impact Assessment (MWIA) is an evidence-based qualitative tool which aims to assess the potential impact of a specific policy, service, project or program on the mental well-being of a population [47]. The MWIA covers three domains that an intervention may have an impact on: [1] ‘Enhancing control’, [2] ‘Increasing Resilience and Community Assets’, and [3] ‘Participation and Social Inclusion’ [47] . Each domain contains evidence-based factors summarised in Table 2. During the MWIA these factors are plotted on a prioritisation grid according to their importance and the impact the intervention was expected to have upon each (see Fig. 1).

**Table 2:** MWIA Evidence-based factors by domains

	Domain		
	Enhancing Control	Increasing resilience and community assets	Participation and social inclusion
Evidence-based factors	A sense of control	Healthy lifestyle	Having a valued role
	Belief in own capabilities and self-determination	Arts and creativity	Activities that bring people together
	Self help	Social networks and relationships	Feeling involved
	Knowledge, skills and resources to make healthy choices	Emotional wellbeing	Accessible and acceptable services
	Opportunities for expressing views and being heard	Ability to understand, think clearly and function socially	Sense of belonging

The MWIA aims to be participatory and inclusive, and is designed to be conducted over a full day. To make the toolkit more appropriate for children the MWIA was shortened to 2 h workshops, conducted pre- and post-intervention. Workshops were facilitated by the research team with a focus on two core activities: 1) defining wellbeing; and 2) plotting factors for the three domains onto grids (see Fig. 1). Activities usually conducted in the MWIA, such as appraisal of social determinants of health and the formulation of recommendations, were felt to be too theoretical and abstract for children to engage with, and were therefore removed. Furthermore, time spent on each activity was reduced to better suit the attention span of children and to fit with schools’ timetables.

The MWIA workshop opened with children developing a group definition of wellbeing, as recommended in the MWIA toolkit [57]. This sought to focus attention of the workshop on defining wellbeing as conceptualised by the participating children. For the factor plotting exercise children were randomly allocated to three groups, one for each domain, and asked to plot the factors for their MWIA domain onto the prioritisation grid (see Fig. 1). Each group was facilitated by a member of the research or intervention team to aid understanding of the exercise. Once completed, prioritisation grids were presented and a plenary discussion was held to establish overall consensus.

#### Quantitative methods

##### Wellbeing check cards

**Fig. 2:**
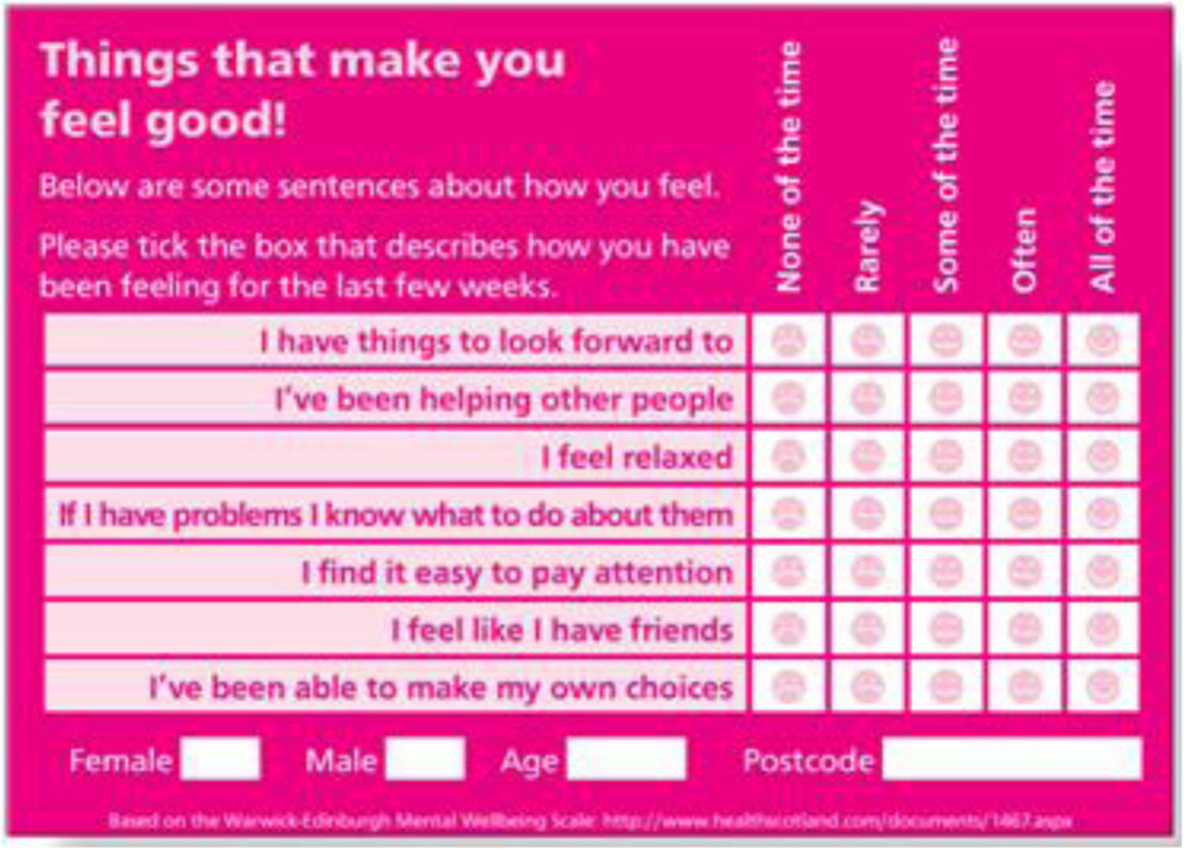
Wellbeing check card

Wellbeing check cards (Fig. 2) were part of the North West PCT evaluation toolkit [48] to measure mental wellbeing in children under 16 years of age. These are based upon the 7-item version of the Warwick Edinburgh Mental Wellbeing Scale which is validated for children over 13 years of age [58]. In this study the self-rated cards were used with all children (aged 9–15) as part of funder efforts to assess their applicability for younger children.

The cards are an anonymous self-reporting tool that uses a Likert scale across seven statements to track individual mental health and wellbeing. The PCT’s adaptation simplified the language and incorporated faces reflecting different emotions in a Likert scale (Fig. 1). The cards were designed to collect data on gender, age and postcode as a means of maintaining anonymity while tracking individual respondents. However, due to the targeted nature of our intervention tracking was not possible because many of the children shared these characteristics.

Participating children were asked to complete the wellbeing check cards during the first and the final gardening session, acting as a pre- post measure. Where this was not possible the cards were completed at the next earliest opportunity and within 1 week of the session.

### Ethics

Ethical oversight of the evaluation was provided by the NHS Trust in which the CAMHS psychotherapist worked. Written or verbal informed consent from children’s parents was required to participate in the intervention and attached evaluation. In line with the participatory approach to evaluation, children also provided their informed assent to participation in the evaluation, gathered at opening MWIA workshops.

### Analysis

Analysis involved separate analysis of the quantitative and qualitative results which are then converged in the discussion to remain in line with the mixed-methods triangulation design [50]. The mean score for the pre- and post-intervention wellbeing check cards were summarised and compared offering an exploratory analysis intended to capture associations in trends only.

As a qualitative tool [59, 60] the MWIA was analysed by two members of the research team who used a thematic coding process deductively driven by the MWIA themes to identify over-arching themes. MWIA findings were triangulated against other qualitative and quantitative findings which reaffirmed their validity. Finally, the research and intervention teams critically reflected on adaptations of the MWIA to assess its fit with evaluating intervention impact upon the Five Ways, and its appropriateness for children aged 9 to 15 years.

## Results

Thirty-six children participated in the Haven Green Space intervention and attached evaluation across three schools. In schools A and B all 12 children invited into the project consented to take part in the intervention and evaluation. In school C, one child chose to opt out after the first session, but had participated in the pre-intervention evaluation session. This child declined to comment about his reasons for opting out. Due to logistical issues (e.g. sickness), between 9 and 12 children from each school were present for each of the pre- and post-evaluation sessions. Table 3 below summarises the gender and social characteristics of the children by school.

**Table 3:** Participant demographics

	Gender	Year group	First Language not English	Refugees / Asylum seekers	Looked after children
School A	6 males 6 females	Year 6 (age 10–11)	1 male 4 females	1 asylum seeker	0
School B	8 males 4 females	Years 5 and 6 (age 9–11)	1 male		1 subject to a CAF^a^
School C	8 males 4 females	Years 7, 8 and 9 (age 11–14)	2 males 3 females	2 with leave to remain status 2 asylum seekers	0

^a^Common Assessment Framework, now called Early Help Assessment Team (EHAT) [61]

Here we present the qualitative findings from the MWIA and the quantitative findings from the Wellbeing Check Cards. In the discussion we also explore how well the MWIA assessed the Five Ways, and reflect upon the tools adaptation and application with children aged 9–15 years.

### Qualitative results: MWIA

The MWIA findings are briefly discussed through comparison across schools, noting differences and similarities relating to the importance and impact of the intervention upon factors when plotted onto prioritisation grids (see Fig. 1 for an example prioritisation grid).

Across all three schools the MWIA captured changes in the relative importance and intervention impact upon each factor. In the domain *enhancing control* the factor “self-help” increased in importance in all three schools, although in school B this remained the least important factor in this domain. The factor “knowledge and skills to make healthy choices” was positively impacted, although in schools C and B it also reduced in importance. In both schools A and B the factor “a sense of control” reduced significantly in importance, whereas in school C this became both more important and was positively impacted by the intervention. Finally, the factor “belief in own capabilities and self-determination” was positively impacted in all three schools, whilst decreasing in importance in Schools A and C.

In the domain *increasing resilience and community assets* notable factors positively impacted by the intervention were “emotional wellbeing” - in School C and A becoming the most important factor - and “social networks and relationships” - which although decreasing in priority for schools A and C consistently increased in intervention impact. In Schools A and B the factor “arts and creativity” became more important whilst intervention impact remained static, whereas in School C this factor significantly decreased in importance whilst increasing in impact. Across all three schools the factor “healthy lifestyle” decreased in importance. This finding corresponds with decrease in the factor “knowledge and skills to make healthy choices” in the domain *enhancing control* which decreased in importance in 2 schools. Finally, the factor “ability to understand, think clearly and function socially” increased in importance and impact in Schools A and C whilst decreasing in school B.

In the *participation and social inclusion* domain a notable finding across all three schools is the factor “having a valued role” which was consistently impacted by the intervention, although it decreased in importance in schools A and B. Similarly, “feeling involved” was consistently impacted, although only increasing in importance in School C. A “sense of belonging” was positively impacted upon in all three schools, with importance remaining static in School C but increasing in schools A and B. The factor, “activities that bring people together” increased in importance in Schools A and B, while in School C this was positively impacted but with no change in importance. Finally, across all three schools the factor “acceptable and accessible services” decreased in priority and remained static in intervention impact.

### Quantitative results: Wellbeing check cards

Scores from the Wellbeing check cards pre- and post-intervention across the three schools were not found to be statistically significant (Table 4). However, a number of findings are worth discussing.

**Table 4:** Wellbeing check card results

	Total score (out of 35)	I have things to look forward to	I’ve been helping other people	I feel relaxed	If I have problems I know what to do about them	I find it easy to pay attention	I feel like I have friends	I’ve been able to make my own choices
School A Pre-intervention	31	4.9 (*n* = 10)	4.8 (*n* = 10)	4.6 (*n* = 9)	4.5 (*n* = 10)	3.9 (*n* = 10)	4.2 (*n* = 10)	4.2 (*n* = 10)
School A Post-intervention	29	4.3 (*n* = 9)	4 (*n* = 9)	4.3 (*n* = 9)	3.8 (*n* = 9)	3.4 (*n* = 9)	4.4 (*n* = 8)	4.5 (*n* = 8)
School B Pre-intervention	30	5 (*n* = 9)	4.5 (*n* = 10)	3.1 (*n* = 9)	3.8 (*n* = 10)	4.4 (*n* = 10)	4.3 (*n* = 10)	4.4 (*n* = 10)
School B Post-intervention	25	3.6 (*n* = 7)	3.3 (*n* = 7)	2.8 (*n* = 6)	3.4 (*n* = 7)	3.6 (*n* = 7)	5 (*n* = 7)	3.6 (*n* = 7)
School C Pre-intervention	28	3.8 (*n* = 11)	3.8 (*n* = 11)	4.2 (*n* = 11)	3.8 (*n* = 11)	3.6 (*n* = 11)	4.5 (*n* = 11)	4.3 (*n* = 11)
School C Post-intervention	31	4.4 (*n* = 8)	4.3 (*n* = 8)	4.9 (*n* = 7)	4.3 (*n* = 8)	4.4 (*n* = 8)	5 (*n* = 7)	4.1 (*n* = 8)

The variable ‘I feel like I have friends’ consistently improved in all three schools. In school C there was general improvement across the statements, except for “I’ve been able to make my own choices”. Conversely, in the primary schools the majority of scores worsen, with the notable exception in School A’s improvement in scores under the statement “I’ve been able to make my own choices”.

## Discussion

This exploratory evaluation of a pilot intervention sought to assess the mental wellbeing of participating children via a mixed-methods triangulation design, and to assess the value of a range of evaluation methods and the “Five ways to wellbeing” evaluation framework. Findings across evaluation methods suggest that there is potential for interventions such as the Haven of Green Space to benefit the mental health and wellbeing of children with BESD. However, given the pilot nature of the intervention, the lack of a control group, and the small number of participating children, it is not possible to draw any firm conclusions regarding the intervention impact upon mental health and wellbeing. Thus, the discussion aims to integrate key findings in the context of trends that we believe warrant further research. We also critically reflect on the adapted evaluation methods and use of the “Five Ways to Wellbeing” evaluation framework.

Positive trends for pro-social behaviour and emotional symptoms are evident in findings from the MWIA. In particular, MWIA factors relating to mental health and wellbeing positively impacted by the intervention in all three schools included “emotional wellbeing” and “self-help”. However, findings from the wellbeing check cards challenge this finding, with worsening scores across many of the domains. The worsening scores may have been influenced by the post assessment coinciding with children’s last days at primary school before moving to secondary school, possibly a bitter-sweet and anxiety provoking time. This is supported by low scores in response to the statement “I feel relaxed”, particularly in School B; and the sharp decrease in scores under the statement “I feel like I have things to look forward to”.

It has been found that children who engage in nature-play have more positive feelings about each other, with natural environments stimulating social interaction [62–64]. Research has furthermore identified that when children play in natural environments their play is more imaginative and creative, in turn promoting language and collaborative skills [65–68], as seen in the “diggy diggy” example in Table 5 (below). These examples similarly represent place-making activities as a location and material setting are imbibed with subjective and emotional attachments through the creation of ritual ceremonies and telling of myths [33–35].

**Table 5:** Application of the Five Ways in Haven Green space intervention

Five Ways Action	Example application in Haven Green Space
Connect: to those around you and to the natural environment	Tea Ceremony: pupils were asked to explore the garden for herbs that could be brewed as herbal tea. The group came together to share tea and discuss issues they were facing in connecting with one another that had been raised when working in the green spaces.
Be active: engage in enjoyable physical activity	All sessions offered the opportunity for physical engagement in gardening, although in line with STH engagement could be either active or passive. In one garden a large root was removed from the centre of the plot, necessitating considerable physical effort by a number of boys who noted satisfaction when the root was removed, including photographing each other holding it as a trophy.
Take Notice: of the world around you and of feelings	In one primary school trees that had been planted were vandalised. This was identified by pupils involved in Haven Green Space who sought teacher support to re-plant them. The pupils led this activity, and discussed with teachers how this vandalism made them feel and why they thought it had been done.
Keep Learning: to build confidence and have fun	All sessions offered learning opportunities, in particular nurturing plants and the natural environment. “Diggy Diggy”: in a primary school one corner of the garden space was devoted to digging. During this activity pupils engaged in creative play involving story telling.
Give: do something nice for a friend or stranger, linking with the wider community	This action was seen in giving time and energy to care for the green spaces, as well as giving plants or the green space itself as an asset to others including friends and family. In one primary school pupils stated that they wanted to create an orchard for future generations of pupils to enjoy and to improve the school grounds for parents, pupils and teachers alike.

Furthermore, findings support the suggestion that Haven Green Space promoted increased pro-social behaviour, for example the positive impacts upon MWIA factors including “feeling involved”, “having a valued role”, “sense of belonging” and “social networks and relationships” suggest increased opportunities for positive social interactions. Similarly, improved scores on the wellbeing check cards in response to “I feel like I have friends” suggest increased pro-social behaviour.

Through engagement in group based activities children learn skills of negotiation and team working whilst iteratively exploring broad health issues through sensory responses to different plants and processes of thriving, wellbeing, and achievement [57]. These experiences have a concomitant impact upon mental health and wellbeing, affecting individual feelings of control, resilience, participation, and inclusion [57]. Haven Green Space as a group based intervention draws on the mental wellbeing and therapeutic benefits of peer interaction and the potential for groups members to be therapeutic agents to each other [69]. Consequently, it is possible that Haven Green Space interacted at multiple levels, especially encouraging engagement and interaction to positively impact children’s feelings of social connectedness and connection to place [34].

Interestingly, MWIA findings suggest that Haven Green Space may have led participants to reassess the primacy of physical health against emotional wellbeing, indicated in consistent decrease in importance of the factors “knowledge and skills to make healthy choices” and “healthy lifestyle”. Whilst the importance of these factors decreased, the positive impact the intervention had upon them remained in all three schools. This may signal a positive repositioning given that the intervention sought to support the mental health and wellbeing of participants rather than promote physical exercise.

Evaluation findings are consistent with theories relating to the positive mental wellbeing impacts of social and therapeutic horticulture. In particular, emphasis upon positive emotional responses to green space correlate with Stress Reduction Theory [18] and emphasis upon spontaneous directed engagement with nature is supported by Attention Restoration Theory [19].

UK government strategies including *No Health without Mental Health* [70, 71] and the Public Health White Paper *Healthy Lives, Healthy People* [72] encourage localised approaches to public health founded upon partnerships that recognise positive mental health as intrinsic to health generally. Having interventions embedded in schools is one form of local partnership working [73], reaching vulnerable children by overcoming traditional barriers to service access [74, 75]. Haven Green Space not only enabled early intervention within the school context, but preliminary findings indicate positive associations with improvements in children’s mental health and wellbeing and social relationships.

### Reflection on the five ways

The Five Ways actions were observed to have been met in the Haven Green Space intervention (Table 5). As the examples in the table suggest, to identify and evaluate the Five Ways in an intervention implemented in a complex social setting presents many challenges. For example, the tea ceremony used to illustrate the action “connect” in School C also stimulated “keep learning”, “take notice”, and “give” as pupils learnt about group dynamics, negotiated conflict resolution, and came to understand the impact of their actions upon others. In light of this complexity it was considered necessary to identify methodologies designed for broad assessment of the impact of this complex intervention upon mental wellbeing.

All methods chosen were felt to assess some or all of the Five Ways. For methods directly involving children focusing upon participatory methodologies [76] was felt to be age-appropriate, complementing the Five Ways approach by reinforcing feedback loops as the children actively reflected upon the intervention to identify the impact it had had upon them. These methods were also felt to be consistent with the group intervention approach [69].

The MWIA as a tool seeks to assess the relative impact of a service or policy upon mental wellbeing in context, and therefore correlates with the aims of the Five Ways which seek to improve mental wellbeing at community, individual and family levels. We felt the MWIA contained factors that explicitly correspond with many of the five ways, for example “Connect” with “social networks and relationships”, and “Be Active” with “healthy lifestyle” (Table 6). In addition, many of the Five Ways actions were implicitly reflected in children’s discussion during the MWIA, for example “Keep learning” in recognising that the intervention aimed to encourage knowledge and skills to respond to mental health and wellbeing.

**Table 6:** Comparison of Five Ways with MWIA

Five Ways	Connect	Be active	Give	Keep learning	Take notice
MWIA	Social networks and relationships	Health Lifestyle	Feeling involved	Understanding the importance of emotional wellbeing	Belief in own capabilities and self determination

The MWIA as a pre- post-measure encourages reflection on potential impacts the intervention may have upon the factors assessed, thereby suggesting areas where feedback loops may be being established. It has been suggested that emotions are a critical component of these feedback loops, with positive emotions acting to signify the benefits of continuing a behaviour and negative emotions the advantage of stopping it [77, 78]. In addition, both the MWIA and the Five Ways explore individual wellbeing from two broad directions: external material and social conditions (i.e. access to services and strength of social networks); and personal resources including emotional and physical health [79].

### Strengths and limitations

Challenges to measuring the relationship between greenspace and mental wellbeing of children have been recognised [80]. This study reinforces the impact of the measurement tool, as well as who is asked – child, parent, or teacher – upon the strength of the association between greenspace and child mental wellbeing, drawing attention to critical issues of research design, whilst supporting approaches that utilise multiple methods and measurements for indicating trends [2]. Recognising these challenges, we reflect on some of the strengths and limitations to the approach adopted in this study.

Although the MWIA method and language were adapted, the use of the adult terminology for MWIA factors raises questions about how these were understood by children. While factors were subjectively defined by pupils or explained by facilitators (where required), this introduces a variable into the way they were understood and applied. Despite these limitations the MWIA clearly resonated with children and we would recommend further efforts at age-appropriate adaptation.

The wellbeing check cards were incorporated by the North West PCT to assess the impact of services. When using this tool there was an impression that the questions relating to how each child had been feeling over “the last few weeks” was not appreciated. Instead, responses were felt to reflect how they felt at that moment, thus were heavily impacted by their experiences in specific sessions. Equally, the timing of their administration during the first and last intervention sessions may have affected participant responses. Consequently, this tool is felt to be limited in its intended ability to capture change over time.

A major strength of this study is that it sought to evaluate a school-based intervention to improve wellbeing, using psychotherapeutic and STH techniques in an innovative way. Children were selected on the basis of exhibiting BESD, living in a deprived neighbourhood, with some experiencing additional external events such as seeking asylum. On this basis the STH programme/intervention would be categorised as targeted (rather than universal or indicated). To our knowledge, this is the first reported evaluation of a targeted STH intervention for children. However, as an uncontrolled pilot pre- post- study findings are preliminary and must be interpreted with caution. Furthermore, the lack of validation of Wellbeing Check Cards, and adaptation of the MWIA, are key methodological weaknesses. Despite these limitations preliminary findings from this pilot study have yielded encouraging results about intervention acceptability and positive trends upon the wellbeing of participating children. In addition, methodological reflections warrant further attention to strengthen the methodological tools to assess such interventions in the future.

## Conclusion

Evaluation findings suggest that group based horticulture activities facilitated by a trained therapist that acknowledge social interaction as an important driver of wellbeing are features of an intervention that are associated with positive trends in the mental and emotional wellbeing of children experiencing BESD. Further research into the potential offered by interventions such as Haven Green Space is required to verify findings from this exploratory pilot study. In relation to research tools and procedures, continued efforts are recommended to develop age-appropriate methods to assess the impact of interventions on children and young people, and continued exploration of how they interact with the Five Ways to Wellbeing as an overarching evaluation framework.

## Abbreviations

BESD: Behavioural Emotional and Social Difficulties
CAF: Common Assessment Framework
CAMHS: Children and Adolescent Mental Health Services
EHAT: Early Help Assessment Team
MWIA: Mental Wellbeing Impact Assessment
OfSTED: Office for Standards in Education
PCT: Primary Care Trust
SDQ: Strengths and Difficulties Questionnaire
SEN: Special Educational Needs
STH: Social Therapeutic Horticulture
WEMWBS: Warwick-Edinburgh Mental Well-being Scale

## Footnotes

1 The Haven Greenspace project was included in a systematic review conducted by Ohly and colleagues (2016), drawing upon an early magazine article reporting preliminary study findings for a teacher audience. Chiumento, A [32]. Haven of Greenspace. Young Minds Magazine. 2012;118(Winter):32–4.. Based on the magazine article Ohly et al. gave the project a poor quality assessment due to failing to meet the reporting standards expected for academic peer review journal articles, standards we believe it was inappropriate to apply to a paper in a practitioner magazine. Recognising this review, we have in this paper sought to ensure this academic publication reports fully on all aspects of the study, including transparent discussion of the study limitations.

2 Puzzled Out is an online CAMHS survey tool: www.puzzledout.com
